# Supplementary material for: Superconducting spintronic heat engine
Source: Nat Commun. 2024 Jun 6;15:4823. doi: 10.1038/s41467-024-49052-z (PMC11156981; doi:10.1038/s41467-024-49052-z)
Supplement: Supplementary file 1 — Supplementary Information [file 41467_2024_49052_MOESM1_ESM.pdf]

Superconducting Spintronic Heat Engine

Supplementary figures

Clodoaldo Irineu Levartoski de Araujo [1,2] Pauli Virtanen [3,*] Maria Spies [1]
Carmen González-Orellana [4] Samuel Kerschbaumer [4] Maxim Ilyn [4] Celia Rogero [4,5] Tero Heikkilä [3] Francesco Giazotto [1] and Elia Strambini [1,*]

[1] *NEST, Istituto Nanoscienze-CNR and Scuola Normale Superiore, I-56127 Pisa, Italy*

[2] *Departamento de Fìsica, Laboratório de Spintrônica e Nanomagnetismo, Universidade Federal de Viçosa, Viçosa,36570-900, Minas Gerais, Brazil*

[3] *Department of Physics and Nanoscience Center, University of Jyväskylä, P.O. Box 35 (YFL), FI-40014 University of Jyväskylä, Finland*

[4] *Centro de Física de Materiales (CFM-MPC), Centro Mixto CSIC-UPV/EHU, 20018 Donostia-San Sebastián, Spain*

[5] *Donostia International Physics Center (DIPC), 20018 Donostia-San Sebastián, Spain*

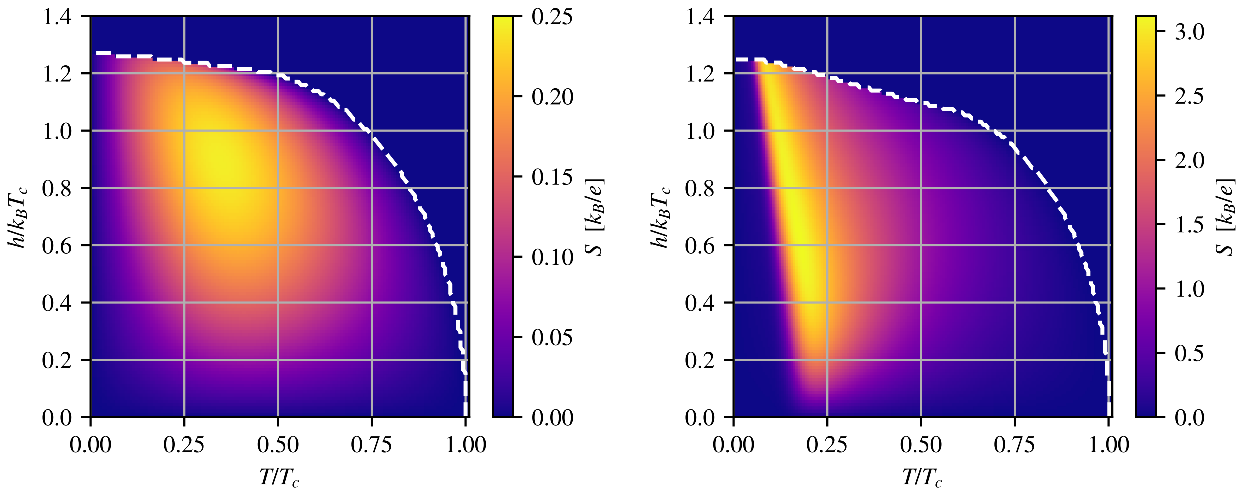

Supplementary Fig. 1: Predicted Seebeck coefficient. S as a function of the exchange field h and temperature T. Left: for the same parameters as in Fig. 2. Right: for $\Gamma_{\text{sf}} = 0$ and small $\hbar\Gamma = 0.05\Delta$. Dashed line indicates the superconducting transition.

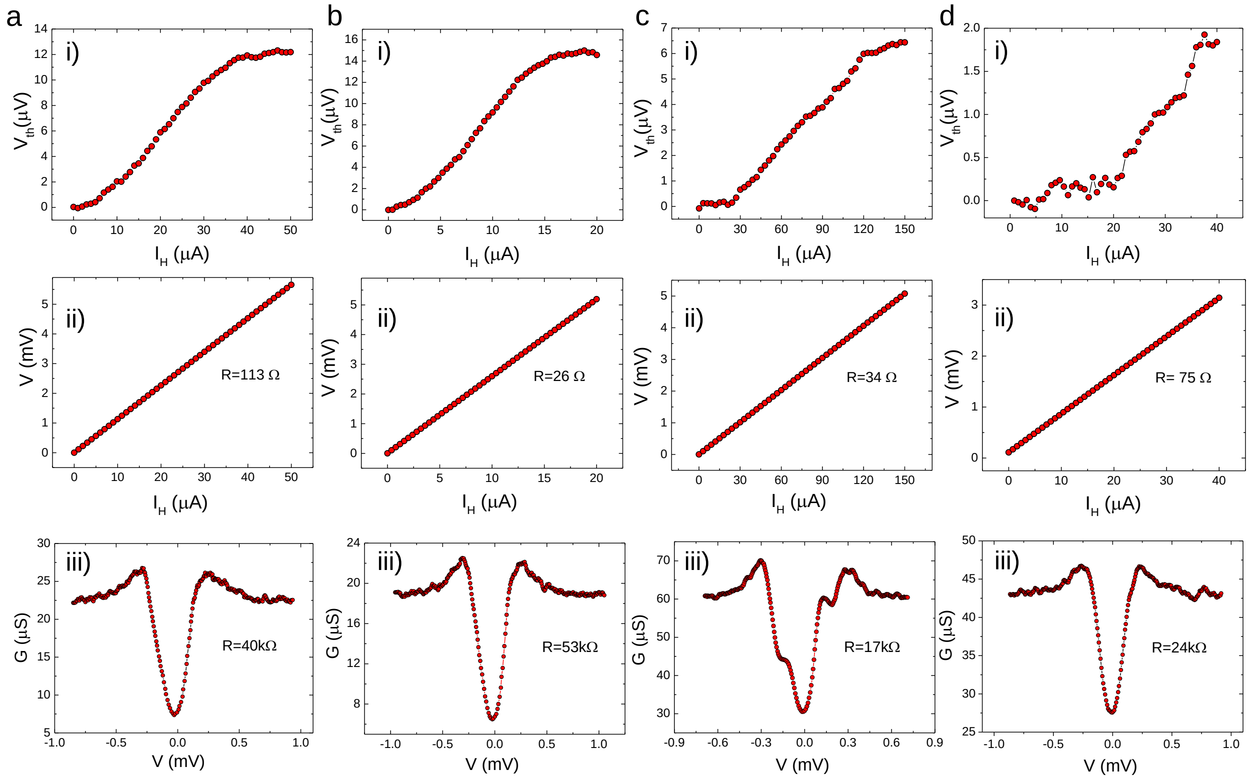

Supplementary Fig. 2: Thermovoltage measurements on different samples. Panels a), b) and c) show measurement on nominally identical samples composed by CaF/Co(14 nm)/AlO$_x$ (5hrs)/Al(20 nm)/EuS(21.5 nm)/SiO2/Si cross bar. Pannel a) is the sample used in the main text. Pannel d) is a similar sample but with the aluminum thickness of 12 nm. i) In the first raw the Thermovoltage V_{th} vs the heating current is compared for the four different samples. The VI characteristics of the cobalt strip and the tunneling conductance of the junctions are presented in rows ii) and iii), respectively. In ii) the strip resistance R is estimated from the slope of the VI. All measurements are performed at $T_{\mathrm{bath}} = 100$ mK and at $B = 10\,\mathrm{mT}$

* pauli.t.virtanen@jyu.fi, elia.strambini@cnr.it

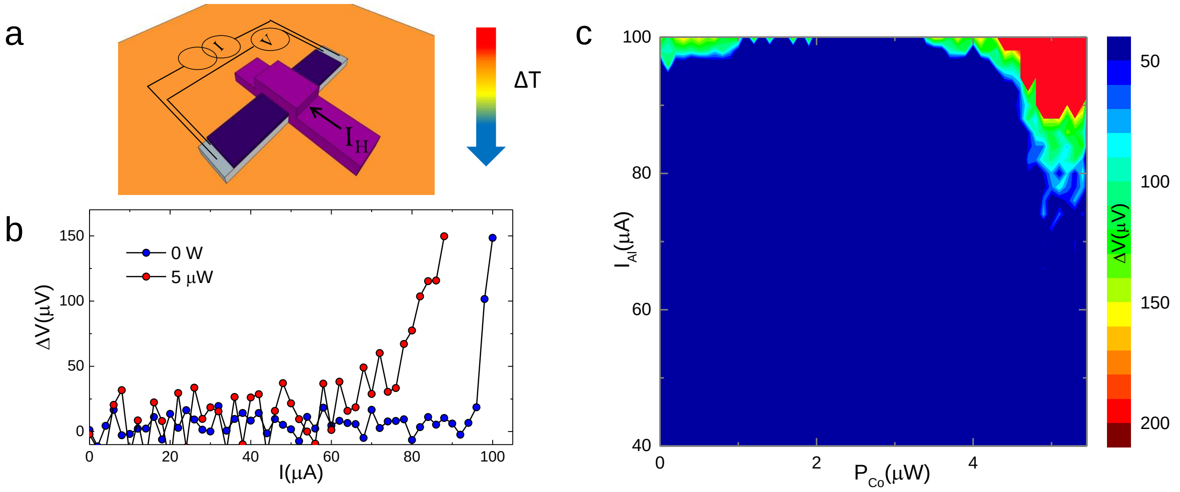

Supplementary Fig. 3: Thermal gradient limit. Critical current in the superconducting aluminum as a function of power injected in the top cobalt strip, as depicted in the cartoon presented in a). b) examples of critical current measurement showing the voltage drop ΔV vs the injected current I for the sample without excitation (blue dots) and under an heating power P_{Co} of $5\,\mu$W injected in the top cobalt strip (red dots). In panel c) we present the $\Delta V(I, P_{\mathrm{Co}})$ showing no change of the critical current for $P_{\mathrm{Co}} < 4\mu\,$W. This suggest the possibility to heat the Co stripe with minimal impact on the temperature of the Al stripe.

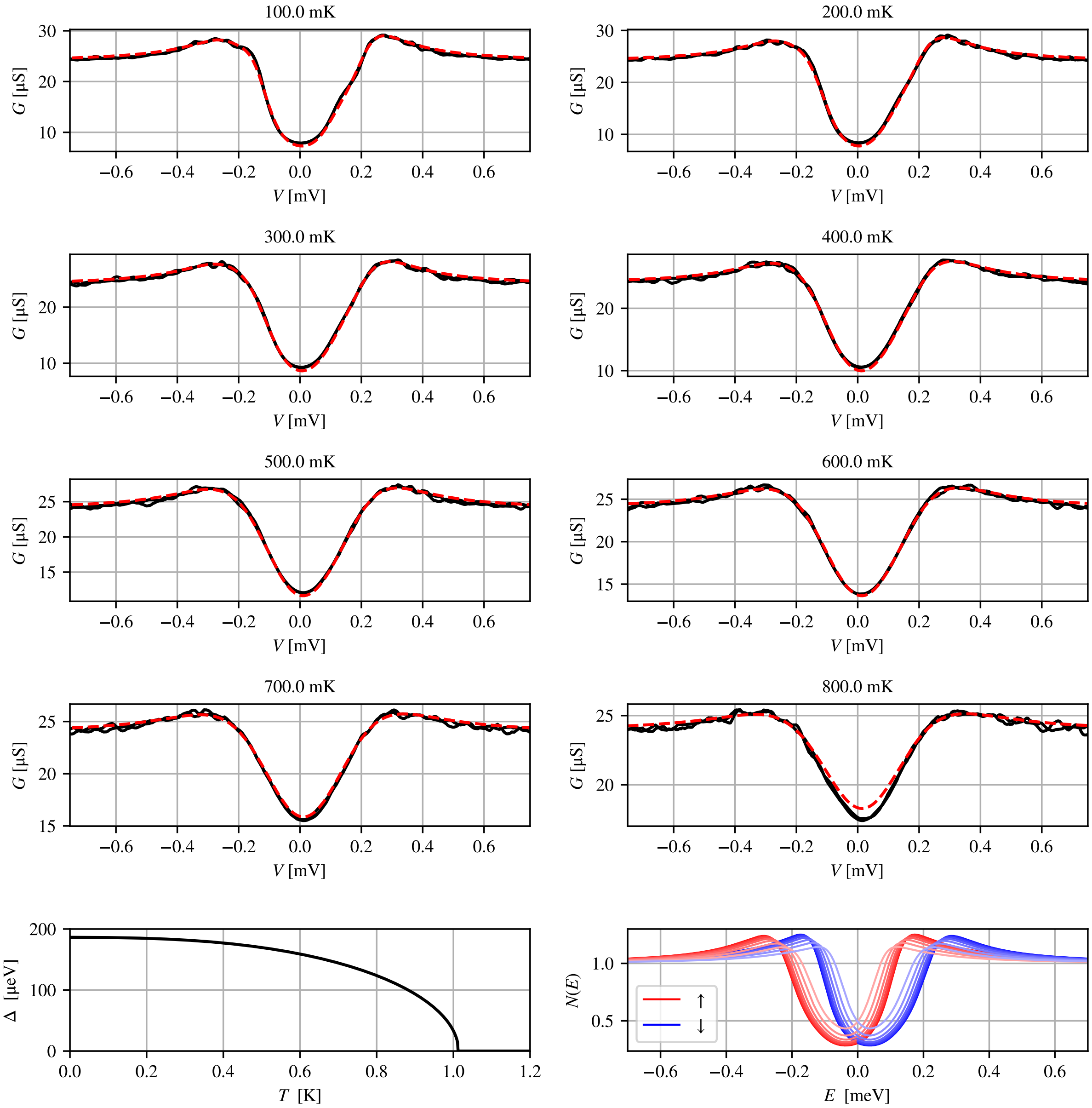

Supplementary Fig. 4: dI/dV **fits for heat engine junction parameters.** Top panels: experimental $G = dI/dV$ data (solid) and theoretical fit (dashed) at different temperatures. Theoretical results are from Eq. 1, and as explained in Methods determine the junction parameters G, Δ_0, h, P, Γ, $\Gamma_{\rm sf}$, assumed independent of temperature and voltage. Bottom panels: corresponding theoretical temperature dependence of Δ, and the normalized spin-dependent density of states $N_{\sigma=\uparrow/\downarrow}(E)$ at the dI/dV temperatures (lighter lines: higher T).
