# Supplementary material for: Superconducting spintronic heat engine
Source: Nat Commun. 2024 Jun 6;15:4823. doi: 10.1038/s41467-024-49052-z (PMC11156981; doi:10.1038/s41467-024-49052-z)
Supplement: Supplementary file 3 — Description of Additional Supplementary Files [file 41467_2024_49052_MOESM3_ESM.pdf]

Description of Additional supplementary files

Supplementary Data 1: The file names of the additional supplementary files refer to the figure of the main text in which the data are reported. If the data are experimental or extracted from simulation the file name reports exp and teo, respectively.
